# Prevalence and Risk Factors of Carotid Plaque Among Middle-aged and Elderly Adults in Rural Tianjin, China

**DOI:** 10.1038/srep23870

**Published:** 2016-03-31

**Authors:** Changqing Zhan, Min Shi, Ying Yang, Hongbo Pang, Shizao Fei, Lingling Bai, Bin Liu, Jun Tu, Yong Huo, Xianjia Ning, Yan Zhang, Jinghua Wang

**Affiliations:** 1 Department of Neurology, Wuhu No. 2 People’s Hospital, Wuhu, Anhui Province, 241000, China; 2 Department of Epidemiology, Tianjin Neurological Institute, Tianjin, 300052, China; 3 Department of Neurology, Tianjin Medical University General Hospital, Tianjin, 300052, China; 4 Department of Cardiology, Peking University First Hospital, Beijing, 100034, China; 5 Department of Ultrasound, Tianjin Huanhu Hospital, Tianjin, 300060, China

## Abstract

Carotid plaque (CP) is associated with cardiovascular and cerebrovascular events. However, population-based studies with a large sample are rare in China, particularly those in the low-income population. We aimed to determine the prevalence of CP and the associated risk factors in the rural areas of northern China. Between April 2014 and June 2014, we recruited 3789 residents aged ≥45 years. B-mode ultrasonography was performed to measure the extent of CP. The prevalence of CP was 40.3% overall, 47.1% in men, and 35.4% in women (P < 0.001). The prevalence of CP increased with increasing age (P < 0.001). The participants with CP were more likely to have hypertension, diabetes, high total cholesterol (TC) levels, and high low-density lipoprotein-cholesterol levels and be a current smoker; however, they were less likely to be obese. Multiple logistic regression analysis, adjusted for confounders, indicated that age, male sex, hypertension, diabetes, current smoking, and high LDL-C levels were the independent risk factors for CP. There was a lower risk of CP with alcohol consumption. The findings suggest that managing the conventional risk factors is crucial to reduce the burden of cardiovascular and cerebrovascular diseases in the low-income population in China.

Cardiovascular disease (CVD), including ischemic heart disease and stroke, is a leading cause of death in both developed and developing countries worldwide^1^, accounting for nearly 42% of all deaths in 2010. Moreover, the 2005–2015 economic burden of CVD in China is estimated to be approximately 550 billion USD^2^,^3^.

Atherosclerosis is the major cause of CVD, and carotid atherosclerosis is associated with an increased risk of CVD and vascular death^4^,^5^,^6^. Stenosis of the internal carotid artery (ICA) is a major risk factor for stroke, with a recurrence rate of 32% at 12 weeks after stroke among patients with symptoms of cerebral ischemia and ≥50% carotid stenosis^7^. Moreover, there is a 2–4% annual risk and 10% 10-year risk of stroke for patients with severe (>70%) carotid stenosis^8^,^9^,^10^,^11^.

Several cohort studies have indicated that carotid plaque (CP) and carotid intima-media thickening (CIMT) are risk factors for future CVD^12^,^13^,^14^ and cerebrovascular diseases^15^,^16^. Furthermore, asymptomatic and preclinical CP is reportedly a better predictor of vascular events than CIMT^15^,^17^,^18^,^19^,^20^,^21^ and can reflect the degree of atherosclerosis^22^,^23^. CP is considered a significant marker on imaging for the future risk of CVD^12^,^24^ and has a high sensitivity for identifying subclinical vascular disease^4^.

Although the associations between CP and CVD risk factors, such as age, sex, hypertension, diabetes, hyperlipidaemia, obesity, smoking status, alcohol consumption, and blood pressure (BP), lipid, and glucose levels have been identified in previous studies^25^,^26^,^27^, data on the associations between CP and CVD risk factors in a population-based in China are limited. Moreover, more than half of the Chinese population lives in rural areas, and they tend to have poor medical insurance, low educational levels, and low income; but large population-based studies among low-income residents are rare.

In the present study, we aimed to determine the prevalence of CP among a low-income population in rural Tianjin, China and to assess the relationships between CP prevalence and the traditional CVD risk factors.

## Results

### Demographic characteristics

Of the 5380 residents aged ≥45 years, 4012 (75%) residents participated in this survey. After excluding 223 residents with a previous history of stroke or myocardial infarction, 3789 participants were included (1560 [41.2%] men and 2229 [58.8%] women).

The age-standardized prevalence of CP was 40.3% overall, 47.1% in men, and 35.4% in women (P < 0.001). The average size of plaque was 22.48 mm (standard error, 0.58 mm), and the median number of lesions was 1 (range 1 to 7). The mean age was 59.92 (9.70) years in the CP group and 61.13 (9.90) years in the non-CP group. The prevalence of CP increased with increasing age (P < 0.001). Significantly fewer years of education and lower BMIs were observed in the CP group than in the non-CP group (P < 0.001 and P = 0.002, respectively). SBP, DBP, and FBG, TC, and LDL-C levels were significantly higher in the CP group than in the non-CP group (all P < 0.05; Table 1).

### Age-standardized prevalence of carotid plaque according to cardiovascular disease risk factors

The participants with CP were more likely to have hypertension, diabetes, high TC levels, and high LDL-C levels and be current smokers, but were less likely to be obese than participants without CP (Table 2). There were no significant differences in alcohol consumption, high TG levels, and low HDL-C levels.

### Risk factors for carotid plaque

The multivariate logistic regression analysis indicated that age (OR, 1.07; 95% CI, 1.05–1.08), male sex (OR, 1.75; 95% CI, 1.40–2.18), hypertension (OR, 1.43; 95% CI, 1.18–1.73), diabetes (OR, 1.81; 95% CI, 1.34–2.44), current smoking (OR, 1.43; 95% CI, 1.11–1.85), and high LDL-C levels (OR, 3.92; 95% CI, 2.70–5.69) were the independent risk factors for CP (Table 3). Lower amount of alcohol consumption was associated with a lower risk of CP, with an OR (95% CI) of 0.64 (0.41–0.99, P = 0.048) for those with alcohol intake <300 g, and 0.57 (0.35–0.94, P = 0.026) for those with alcohol intake 300–500 g.

## Discussion

This report describes the prevalence of and relevant risk factors for CP in the low-income population in China based on a large population-based study, resulting in an overall CP prevalence of 40.3% and a significantly higher prevalence in men (47.1%) than in women (35.4%). In addition to the male sex, older age, hypertension, diabetes, current smoking, and high LDL-C levels were risk factors for CP, whereas alcohol consumption was protective.

Of the few reports that have described population-based studies of the prevalence of CP, the Northern Manhattan Cohort Study (NOMAS), which was a population-based cohort study with a unique race/ethnic distribution of community residents aged ≥39 years, reported CP prevalence of 57% overall, 70% in Caucasian participants, 52% in Hispanic participants, and 58% in black participants^28^. In Beijing, China, the prevalence of CP was 60.3% among urban residents aged 43–81 years, almost 70% in the elderly aged ≥60 years, and 80% in the elderly aged ≥70 years^29^. The overall prevalence of CP in the present study of a low-income population was lower than in these previous studies, as were the prevalence in the participants aged 65–74 years (53.9%) and ≥75 years (69.5%). The ethnic diversity or socioeconomic status might explain these differences.

The risk factors for CP in the present study were older age, male sex, hypertension, diabetes, current smoking, and high LDL-C levels, while lower dose alcohol consumption was associated with a lower risk of CP; these findings are supported by those of previous studies. Age is considered an important risk factor for atherosclerotic plaque, and a positive relationship between the prevalence of CP and age has been reported previously^30^. Moreover, the prevalence of CP is higher in men than in women^31^. While hypertension and diabetes have been significantly associated with CP^12^,^32^,^33^,^34^, LDL-C might have the strongest relation with CP^35^. The risk of CP was 3.9 times higher with high LDL-C levels than with the levels in the reference group. Oxidized LDL-C can enter and accumulate within the arterial walls and is involved in the inflammatory process in atherosclerosis^36^. Therefore, these conventional risk factors might contribute to CP by inducing endothelial dysfunction, hyperinsulinaemia, hemodynamic stress, and multiple metabolic alterations^37^,^38^,^39^.

The risk of CP in the present study was 36% and 43% lower with a lower amount of alcohol consumption. Alcohol inhibited the progression and initiation of atherosclerotic lesions in mice^40^. The underlying mechanism might involve the inhibitory effects of ethanol on fatty acid oxidation and attenuation of increased lipid synthesis^41^.

There were several limitations in this study. First, the study population was from a local town in Tianjin, China, there was the limited representation. Second, the design of cross-section study may have led to a selection bias, especially among those healthy elderly. However, those patients with the previous histories of cardiovascular disease and cerebrovascular disease were excluded in this study, all participants were asymptomatic. This may decrease the bias.

## Conclusions

This study was the cross-sectional on the prevalence of CP in a low-income population in China. In this study involving a middle-aged and elderly rural population in northern China, the age-standardized prevalence of CP was 40.3%, which is lower than that reported in developed countries and urban populations. This may be associated with the race and life-style, which is needed to researched further. Older age, male sex, hypertension, diabetes, current smoking, and high LDL-C levels were independent risk factors for CP, whereas a lower amount of alcohol consumption was protective. Therefore, managing these conventional risk factors in low-income populations in China could reduce the burden of CVD and cerebrovascular diseases.

## Materials and Methods

### Participants and study design

This study was performed between April 2014 and January 2015, with the study population from the Tianjin Brain Study^42^,^43^,^44^,^45^. In brief, the total population included 14251 persons distributed within 18 administrative villages. Approximately 95% of the residents were low-income farmers. The main source of income was grain production in this area, and the per capita disposable income (an individual’s ability to purchase goods or services) was <1600 US in 2014^46^. In 2011, the average length of education was 5.26 years.

All residents aged ≥45 years without a history of cardiovascular and cerebrovascular diseases from the Tianjin Brain Study were eligible for this study, but those with a history of or current symptomatic cardiovascular and cerebrovascular diseases were excluded.

Demographic information, previous medical history, family history of disease, and behavioural factors were collected using a predesigned questionnaire. A physical examination and assessment of fasting glucose and lipid levels were performed at the same time.

All investigative protocols were approved by the ethics committee of Peking University First Hospital; the methods were carried out in accordance with the approved guidelines, and informed consent was obtained from each participants.

### Survey for risk factors

The surveys were conducted through face-to-face interviews by trained research staff to collect name; sex; date of birth; educational level; previous history of hypertension, diabetes mellitus, stroke, transient ischemia, and coronary heart disease; family history of hypertension, diabetes mellitus, stroke, and coronary heart disease; cigarette smoking (≥1 cigarette per day for ≥1 year); and alcohol consumption (drinking alcohol ≥1 time per week for 1 year).

### Physical examinations

BP, height, and weight were measured. Body mass index (BMI) was calculated as weight (kg) divided by the square of height (m^2^). Serum fasting blood glucose (FBG), total cholesterol (TC), triglyceride (TG), high-density lipoprotein cholesterol (HDL-C), and low-density lipoprotein cholesterol (LDL-C) levels were measured and analysed at the central laboratory of Tianjin Medical University General Hospital. A carotid ultrasonography examination and 12-lead echocardiography were also performed.

### Ultrasonography measurements

One trained technician blinded to participants’ information performed all the ultrasound exams. The patients were examined in the supine position using B mode ultrasonography (Terason 3000; Burlington, MA, US) with a 5–12 MHz linear array transducer. Extracranial carotid artery trees (common carotid artery, the bifurcation, internal and external carotid artery) on both sides were screened for plaque. Images were obtained and digitally stored according to a standard protocol. Both longitudinal and transvers dynamic images of each plaque were stored.

### Survey Procedure

Local village doctors informed all qualified residents door-to-door according to a predefined procedure one day before examination. We performed physical examination (including blood pressure, weight, and height measurement, carotid ultrasonography, and 12-lead echocardiography examination) and blood sample collection at local village clinics between April 15, 2014 and June 30, 2014. All blood samples were sent to the central laboratory at Tianjin Medical University General Hospital for measurement of total cholesterol, triglyceride, high-density lipoprotein cholesterol, and low-density lipoprotein cholesterol levels within 12 hours of collection, and to the central laboratory at Tianjin Ji County People’s Hospital for measurement of fasting blood glucose levels within 2 hours of collection. Measurement of carotid plaque and IMT was performed by one practiced technician between July 1, 2014 and January 8, 2015.

### Definitions

Hypertension was defined as systolic BP (SBP) ≥140 mm Hg or diastolic BP (DBP) ≥90 mmHg or taking medication for hypertension. Diabetes was defined as FBG ≥7.0 mmol/L or taking medication for diabetes. Obesity was defined as a BMI ≥28.0 kg/m^2^, and overweight was defined as a BMI of 24.0–27.9 kg/m^2^ 47.

High FBG was defined as ≥6.1 mmol/L^48^. High TC was defined as ≥6.22 mmol/L. High TG was defined as ≥2.26 mmol/L. High LDL-C was defined as ≥4.14 mmol/L, and low HDL-C was defined as ≥1.04 mmol/L^49^.

Plaques are focal structures that encroach into the arterial lumen by at least 0.5 mm or 50% of the surrounding IMT, or demonstrate a thickness of >1.5 mm, as measured from the intima-lumen interface to the media adventitia interface^50^. Subjects with carotid plaque were definite as present of one ≥lesions, no matter the numbers of carotid plaque.

### Statistical analyses

All participants were categorized based on the presence of CP into the CP and non-CP groups. Continuous variables are presented as mean and standard deviation and were compared between the groups using Student’s *t*-tests. Categorical variables are presented as frequencies and 95% confidence intervals (CIs) and were compared using Chi-square tests. The age-standardized prevalence of CP was calculated dividing the population into 10 age groups with the direct method using the world standard population: <35, 35–39, 40–44, 45–49, 50–54, 55–59, 60–64, 65–69, 70–74, and ≥75 years^51^. Associations between CP (binomial dependent variable) and CVD risk factors (independent variables) were determined using univariate and multivariate logistic regression analyses, and the results are presented as unadjusted odds ratios (ORs) and 95% CIs or adjusted ORs and 95% CIs, respectively. Of these CRFs, age and education level were assessed as continuous variables, and history of hypertension and diabetes as binomial variables. BMI and smoking and drinking status were evaluated by categorized variables. BMI was categorized as normal weight, overweight, and obesity, with normal weight as reference; smoking status was divided into never smoking, ever smoking, and current smoking, with never smoking as reference; alcohol consumption was divided into never drinking, ever drinking, current drinking level 1 (alcohol consumption per week <300 g), current drinking level 2 (amount of alcohol consumption per week 300–500 g), current drinking level 3 (amount of alcohol consumption per week 501–750 g), and current drinking level 4 (amount of alcohol consumption per week >750 g) according to the quartile of alcohol consumption amount per week, with never drinking as the reference. A P value <0.05 was considered statistically significant. SPSS for Windows (version 13.0; SPSS Inc., Chicago, IL, USA) was used for analyses.

## Additional Information

**How to cite this article**: Zhan, C. *et al.* Prevalence and Risk Factors of Carotid Plaque Among Middle-aged and Elderly Adults in Rural Tianjin, China. *Sci. Rep.* **6**, 23870; doi: 10.1038/srep23870 (2016).

## Figures and Tables

**Table 1:** Demographic characteristics of the participants, based on the presence of carotid plaque (CP).

Characteristics	CP	Non-CP	P
Total, n (%)	1574 (41.5)	2215 (58.5)	—
Men	782 (50.1)	778 (49.9)	<0.001
Women	792 (35.5)	1437 (64.5)	
Age, year, mean (SD)	63.38 (9.49)	61.13 (9.90)	<0.001
Age group, n (%)			<0.001
45 ~ 54 years	281 (22.7)	955 (77.3)	
55 ~ 64 years	684 (45.2)	830 (54.8)	
65 ~ 74 years	390 (53.9)	334 (46.1)	
≥75 years	219 (69.5)	96 (30.5)	
Education, year, Mean(SD)	4.91 (3.78)	5.69 (3.61)	<0.001
SBP, mean(SD), mmHg	151.58 (23.25)	142.76 (20.60)	<0.001
DBP, mean(SD), mmHg	87.32 (11.56)	86.45 (11.28)	0.021
BMI, mean(SD), Kg/m^2^	25.35 (3.70)	25.72 (3.67)	0.002
FBG, mean(SD), mmol/L	6.09 (1.83)	5.81 (1.34)	<0.001
TC, mean(SD), mmol/L	4.99 (1.15)	4.78 (1.04)	<0.001
TG, mean(SD), mmol/L	1.76 (1.24)	1.76 (1.31)	0.903
HDL-C, mean(SD), mmol/L	1.45 (0.45)	1.46 (0.47)	0.582
LDL-C, mean(SD), mmol/L	3.07 (1.44)	2.43 (1.02)	<0.001

SD, standard deviation; SBP, systolic blood pressure; DBP, diastolic blood pressure; BMI, body mass index; FBG, fasting blood glucose; TC, total cholesterol; TG, triglycerides; HDL-C, high density lipoprotein cholesterol; LDL-C, low density lipoprotein cholesterol.

**Table 2:** The age-standardized prevalence of carotid plaque by cardiovascular disease risk factor*.

Risk factors	Yes	No	P
Hypertension	43.53 (0.98)	33.64 (1.36)	<0.001
Diabetes	49.60 (2.17)	38.78 (0.86)	<0.001
BMI groups:			0.030
Normal weight	40.97 (1.36)	—	
Overweight	39.47 (1.22)	—	
Obesity	40.25 (1.65)	—	
Smoking status:			<0.001
Never smoking	37.05 (0.91)	—	
Ever smoking	36.43 (3.66)	—	
Current smoking	50.58 (1.79)	—	
Alcohol consumption:			0.112
Never drinking	39.59 (0.86)	—	
Ever drinking	52.89 (7.13)	—	
Current drinking	43.34 (3.24)	—	
High TC	47.12 (2.53)	39.32 (0.85)	0.001
High TG	40.36 (1.72)	39.93 (0.91)	0.447
Low HDL-C	41.61 (2.11)	39.22 (0.87)	0.478
High LDL-C	66.73 (2.74)	37.86 (0.83)	0.001

TC, total cholesterol; TG, triglycerides; HDL-C, high density lipoprotein cholesterol; LDL-C, low density lipoprotein cholesterol.

^*^all data was presented as rate (%) with standard error of rate.

**Table 3:** Logistic regression analysis for the presence of carotid plaque based on the presence of cardiovascular disease risk factors.

Risk factors	Reference	Adjusted OR (95%CI)	*p*
Age	—	1.07 (1.05, 1.08)	<0.001
Gender:			
Men	Women	1.69 (1.35, 2.11)	<0.001
Education	—	1.01 (0.98, 1.03)	0.623
Hypertension	Non-hypertension	1.41 (1.16, 1.71)	<0.001
Diabetes	Non-diabetes	1.47 (1.17, 1.86)	0.001
BMI:			
Overweight	Normal weight	0.88 (0.72, 1.07)	0.186
Obesity	Normal weight	0.88 (0.70, 1.12)	0.309
Smoking status:			
Ever smoking	Never smoking	0.86 (0.58, 1.29)	0.475
Current smoking	Never smoking	1.45 (1.11, 1.88)	0.006
Alcohol drinking status:			
Ever drinking	Never drinking	1.25 (0.59, 2.68)	0.560
Current drinking (L1)	Never drinking	0.64 (0.41, 0.99)	0.048
Current drinking (L2)	Never drinking	0.57 (0.35, 0.94)	0.026
Current drinking (L3)	Never drinking	0.72 (0.42, 1.24)	0.239
Current drinking (L4)	Never drinking	0.80 (0.51, 1.25)	0.324
High TC	Normal TC	0.77 (0.55, 1.08)	0.130
High TG	Normal TG	1.01 (0.82, 1.25)	0.910
Low HDL-C	Normal HDL-C	0.90 (0.70, 1.14)	0.365
High LDL-C	Normal LDL-C	3.92 (2.70, 5.69)	<0.001

OR, odds ratio; CI, confidence interval;

L1, amount of alcohol consumption per week <300 g; L2, amount of alcohol consumption per week 300–500 g; L3, amount of alcohol consumption per week 501–750 gram; L4, amount of alcohol consumption per week >750 g.

TC, total cholesterol; TG, triglycerides; HDL-C, high density lipoprotein cholesterol; LDL-C, low density lipoprotein cholesterol.
